# Editorial: Neuropsychology Through the MRI Looking Glass

**DOI:** 10.3389/fneur.2020.609897

**Published:** 2020-11-12

**Authors:** Ovidiu Lungu, Martin Bares

**Affiliations:** ^1^Department of Psychiatry, University of Montreal, Montreal, QC, Canada; ^2^Centre de Recherche de l'Institut Universitaire de Gériatrie de Montréal, Montreal, QC, Canada; ^3^McConnell Brain Imaging Centre, Montreal Neurological Institute, McGill University, Montreal, QC, Canada; ^4^First Department of Neurology, Faculty of Medicine, Masaryk University and St. Anne's University Hospital, Brno, Czechia; ^5^Department of Neurology, School of Medicine, University of Minnesota, Minneapolis, MN, United States

**Keywords:** neuropsychology, MRI, fMRI—functional magnetic resonance imaging, resting-sate fmri, PET—positron emission tomography

Since its inception as a science, psychology has been explicitly preoccupied with the mind-body relationship, specifically with the role that the nervous system plays in shaping how we perceive the world around us and how we react in our interaction with it. The evidence in this regard are the second and third chapters of William James's 1890 “The Principle of Psychology” (Volume 1) (1), which explain the functions of the brain and the general conditions underlying its activity, as well as the entire work of Wilhelm Wundt's 1904 “Principles of Physiological Psychology” (2), which explicitly linked the evolution of various mental functions to the organization and physiology of the nervous system. Yet, it was not until 1980s when the neuropsychology has established itself as a branch of psychology in its own right, aiming to explain how the brain and the other parts of the nervous system influence one's cognition, emotions and behaviors using a variety of methods from observation and questionnaires to computerized tasks gathering reaction time and electrophysiology. The knowledge thus obtained in healthy individuals was then employed by neuropsychologists in assessing the impact of various diseases on brain structure and function. The next decade, the 1990s, ushered in the neuroscience era, with the development of various techniques of magnetic resonance imaging (MRI), of which the functional MRI (fMRI) proved to be a key tool in uncovering, *in vivo*, the neurophysiological substrate of specific brain functions. Neuropsychologists quickly incorporated these new techniques in their research methods arsenal and nowadays they are part of the regular curricula in most neuropsychology training programs. In the current Research Topic we sought to gather a group of articles that will showcase the use of various MRI techniques in neuropsychology in both healthy individuals and those affected by different diseases, thus highlighting the specific benefits that MRI can bring in this field.

This Research Topic includes 12 articles that showcase both anatomical (Czekóová et al.; Hlavatá et al.; Liu et al.) and functional (Dash et al.; Fajnerova et al.; Gabitov et al.; Potvin et al.; Qin et al.; Sojka et al.; Solstrand Dahlberg et al.) MRI methods, as well as positron emission tomography (PET) (Longarzo et al.; Trošt et al.), in investigations that range from a case report (Longarzo et al.), to experimental paradigms that involved groups of participants (Czekóová et al.; Dash et al.; Fajnerova et al.; Gabitov et al.; Hlavatá et al.; Qin et al.; Sojka et al.; Trošt et al.), to reviews and meta-analyses (Liu et al.; Potvin et al.; Solstrand Dahlberg et al.). Likewise, the studies included in this special issue sought to investigate the neurophysiological substrates of various brain functions in both healthy individuals and those afflicted by diseases.

Regarding the use of anatomical MRI in neuropsychology, we showcase here four articles. One of these articles is a case report by Longarzo et al. of a 67-year-old male patient with bilateral thalamic stroke who was investigated using positron emission tomography, magnetic resonance imaging (tractography), and cognitive assessments, performed at baseline and at two follow-up evaluations at 6 and 18 months post-injury. Interestingly, the authors also included 13 healthy individuals matched for age and gender, who followed the MRI protocols of the study. The study presents three major findings: an association between structural and metabolic changes within the fronto-thalamic circuitry in the patient, significant differences between the patient and controls involving the anterior thalamic radiation, one of the major fiber tracts in the fronto-thalamic circuitry and—importantly for clinicians—adaptations to bilateral vascular thalamic injury at multiple levels, revealing the metabolic, functional, and microstructural alterations attending to multidomain neuropsychological impairment. Two articles investigated brain structural differences between patients and their healthy counterparts in conjunction with the results of various clinical neuropsychological assessments. Hlavatá et al. report structural differences in cortical areas related to impulse control in Parkinson's disease patients with and without impulse control disorders (ICD) relative to healthy controls. Moreover, patients without ICD had lower volumes and cortical thickness of bilateral inferior frontal gyrus, whereas those with ICD presented higher volumes of right caudal anterior cingulate and rostral middle frontal cortex. Interestingly, patients with ICD performed no worse than healthy controls in various behavioral tasks previously hypothesized as robust impulsivity measures, thus cautioning against quick interpretation of behavioral tests without additional physiological investigations. Czekóová et al. examined the association between gray matter (GM) atrophy in multiple sclerosis (MS) patients and their performance on tasks measuring several fundamental components of social cognition. They report that patients had slower processing speed, poorer perspective taking, and less imitation compared to healthy controls and that these impairments were associated with GM atrophy in putamen, thalami, and anterior insula, predominantly in the left hemisphere. The last paper in this group comes from a new research area in neuroscience dealing with the microbiota-gut-brain axis. Liu et al. conducted a scoping review of recent studies of healthy individuals and patients with diverse neurological disorders that employed a combination of advanced neuroimaging techniques and gut microbiome analyses. The authors report that gut microbiota profile is significantly associated with markers of brain microstructure, but also with those related to functional intrinsic neural activity and brain connectivity at-rest. These findings highlight the need for longitudinal studies that include assessments of the gut microbial community structure and microbial metabolomics in conjunction with neuroimaging and behavioral testing in order to parse out the directionality and causality within the microbiota-gut-brain axis.

Two articles included PET as imaging technique. One is the case report of the 67-year-old male patient with bilateral thalamic stroke that was already mentioned above (Longarzo et al.). The other is a systematic review of studies that used PET and [^18^F]-fluorodeoxyglucose (FDG-PET) to investigate changes in brain metabolism in Parkinson's Disease and other α-synuclein pathologies (Trošt et al.). In this review, Trošt et al. conclude that the neuropsychological data in PD and related α-synucleinopathies correlate with metabolic neuroimaging data, thus suggesting that the latter may be used to derive biomarkers of disease progression.

While the remaining seven articles share the fMRI as their main neuroimaging technique, they can be clustered in two main groups based on whether they investigated the brain activity at rest or during tasks.

The group that used resting-state fMRI (rs-fMRI) contains two articles, one assessing the changes in functional connectivity in patients with obsessive-compulsive disorder (OCD) relative to healthy controls and in conjunction with other variables assessed with neuropsychological tests (Fajnerova et al.) and the other investigating the topological organization of different brain networks in patients with type 2 diabetes mellitus (T2DM), again relative to healthy controls and in association with cognitive dysfunction assessed by neuropsychological tests (Qin et al.). In the first article, Fajnerova et al. report altered functional connectivity patterns in OCD patients relative to their healthy counterparts in association with the severity of cognitive and clinical symptoms. In the second article, Qin et al. found changes in topologic properties of several brain networks in T2DM patients relative to healthy controls that were correlated with cognitive performance.

Of the remaining five articles, three used task-related fMRI in original investigations (Dash et al.; Gabitov et al.; Sojka et al.) and two were meta-analyses of original studies that employed the same neuroimaging technique (Potvin et al.; Solstrand Dahlberg et al.). Sojka et al. investigated the brain activity of patients with functional movement disorder (FMD) and matched healthy controls during an emotion regulation task. They report increased activation in FMD patients in several brain areas when observing negative pictures, especially in areas associated with self-referential processing in voluntary emotional regulation and lower emotional awareness. Dash et al. examined the effect of age and bilingualism on the brain networks underlying attentional processes. The authors found an increase in brain activity in the frontal and parietal areas in elderly when compared to young bilinguals, as well as a negative correlation between the proficiency level in the second language and activation level in frontal areas, which was observed only in the elderly group, thus providing evidence for age-related neuroplasticity. Gabitov et al. investigated local and long-distance changes in functional connectivity between areas involved in the acquisition of a motor skill during the course of motor learning in a group of healthy individuals. The authors report task-induced changes in functional connectivity that reflect reconfiguration of the intrinsic connectivity patterns within the somatomotor network during learning, changes that cannot be predicted or detected using the traditional brain activation contrasts.

The last two articles are meta-analyses. In the first article, Potvin et al. conducted a systematic review of schizophrenia literature in order to test the hypothesis that self-disturbances in schizophrenia may arise from impaired activity in the cortical midline structures and the temporoparietal junction. The authors report that the meta-analysis provided partial support for this hypothesis, with decreased activations only in the dACC and dorsomedial prefrontal cortex, which are involved in cognitive control and/or salience attribution, as well as decision-making, respectively. Given the unexpected decreases in activity in thalamus, which is not a core region of the default-mode network, and is involved in information integration, the authors suggest that the thalamic alterations may compromise self-coherence in schizophrenia. In the second article, Solstrand Dahlberg et al. examined the current fMRI PD literature with the goal of parsing out the contribution of the cerebellum in motor and non-motor functions in this disease. The results revealed that the main cerebellar implications in PD is linked to cognitive functioning, with no significant differences being observed in this structure between PD patients and healthy controls in studies using motor paradigms. Nevertheless, the meta-regression using only data from PD patients indicated that there was a negative correlation between disease severity and cerebellar activation during motor paradigms despite a lack of correlation with disease duration. The authors suggest that these results suggest the presence of a compensatory mechanism to the dysfunctional basal ganglia in PD, where cerebellum may be employed to cope with motor demands.

We believe that the collection of articles grouped in this special issue will increase our awareness of the usefulness of various MRI methods in different areas of neuropsychology. In our opinion, one of the main uses of MRI in neuropsychology is to provide a physiological basis and validation for neuropsychological assessments that are employed in clinical practice. The heterogeneity of the articles in this Research Topic should not be viewed as a feeble, but rather as a strong feature of the research in this field. It highlights the advantages of using the same hardware solution (the MRI scanner), combined with the flexibility of employing various MRI sequences (i.e., structural, functional), data analysis techniques (i.e., model-based, data-driven) and experimental paradigms (i.e., task-based, resting-state) to answer a large diversity of research questions, both fundamental and clinical.

## Author Contributions

OL and MB wrote the editorial article. Both authors contributed to the article and approved the submitted version.

## Conflict of Interest

The authors declare that the research was conducted in the absence of any commercial or financial relationships that could be construed as a potential conflict of interest.
